# Inferomedial cortical bone contact and fixation with calcar screws on the dynamic and static mechanical stability of proximal humerus fractures

**DOI:** 10.1186/s13018-018-1031-7

**Published:** 2019-01-03

**Authors:** Xiaofeng Zhang, Junwu Huang, Lin Zhao, Yi Luo, Hanxin Mao, Yanfeng Huang, Weibing Chen, Qi Chen, Bangjun Cheng

**Affiliations:** Department of Orthopedics, Shanghai Sixth People’s Hospital, Jinshan Branch, Shanghai, China

**Keywords:** Proximal humeral fracture, Locking plate, Inferomedial cortical bone, Calcar screw, Biomechanics, Static and dynamic stability

## Abstract

**Background:**

This study aimed to explore the effect of retaining inferomedial cortical bone contact and fixation with calcar screws on the dynamic and static mechanical stability of proximal humerus fractures treated with a locking plate.

**Methods:**

Twelve Synbone prosthetic humeri (SYNBONE-AG, Switzerland) were used for a wedge osteotomy model at the proximal humerus, in four groups. In the cortex contact + screw fixation group and cortex contact group, the inferomedial cortical bone contact was retained. In the screw fixation group and control group, the inferomedial cortical bone contact was not retained. Calcar screw fixation was implemented only in the screw fixation groups. The dynamic and static mechanical stability of the models were tested with dynamic fatigue mechanics testing, quasi-static axial compression, three-point bending, and torsion testing.

**Results:**

The cortex contact + screw fixation group showed the longest fatigue life and the best stability. There was 35% difference in fatigue life between the cortex contact + screw fixation group and the cortex contact group, 43%between the cortex contact + screw fixation group and screw fixation group, and 63% between the cortex contact + screw fixation group and screw fixation group (*P* < 0.01). The cortex contact + screw fixation group showed the best axial compressive stiffness, bending stiffness, and torsion stiffness; these were successively decreased in the other three groups (*P* < 0.01).

**Conclusion:**

Retaining inferomedial cortical bone contact and fixation with two calcar screws maintained fracture stability with the highest strength and minimum deformation. Of the two methods, restoration of the inferomedial cortical bone support showed better dynamic and static biomechanical properties than placement of calcar screws alone.

## Background

Proximal humeral fractures commonly occur, often in elderly patients after a fall, where they are the third most common osteoporotic fracture [1]. In most cases, these fractures are minimally displaced and are effectively treated with conservative treatment. But moderate or severe displacement may require surgical intervention [2]. Interlocking intramedullary nails or percutaneous fixation with locking plate techniques are routinely used in the treatment of proximal humeral fractures [3, 4]. Relatively speaking, the interlocking intramedullary nail tends to have more complications and higher technical requirements, so that lateral long-type proximal humeral internal locking plate (PHILP) is often used, with satisfactory results [5]. PHILP has achieved satisfactory results in fixation of proximal humeral fractures. Nevertheless, a number of invalid fixations have also been reported [5–7]. One of the key issues is whether the fracture fixation retains contact with the inferomedial cortical bone and whether to insert two calcar screws along the tangential direction of the inferomedial cortex to strengthen the stability of the fracture and prevent fracture introversion [8]. Locking screws are thought to improve fixation of the head and soft metaphyseal, especially with osteoporotic bone, which are frequently associated with patients who experience these fractures [9].

It is important during minimally invasive surgery to avoid iatrogenic radial nerve injury, and PHILP with a spiral plate is likely to achieve more satisfactory results [10]. A spiral long-type PHILP locking plate can avoid point stripping on the deltoid and interference to radial nerve and has better static biomechanical properties. The importance of the calcar screws in minimally invasive surgery is debated [11, 12]. The humeral calcar refers to the inferomedial cortical area where the humeral head extends to the surgical neck of the humerus. Morphological and microstructural analysis of the proximal humerus shows that this area is the best in terms of the thickness and density of the cortical bone. Inserting one or two screws along the tangential direction of the inferomedial cortex to enhance the stability of the fracture can prevent the varus of the fracture. This screw is called the calcar screw [13].

There has been particular controversy over the treatment of comminuted fractures of the proximal medial humerus [13]. These fractures are associated with re-displacement and with reduction relying on the inferomedial cortical bone at the proximal humerus and calcar screw fixation [14]. Anatomic reduction and firm fixation of the humerus is the basic guarantee of proximal stability [15]. However, while there are many clinical studies that show follow-up data [16–18], with useful recommendations for the management of complex proximal humeral fractures [13], few studies have been undertaken on the dynamic and static stability of different methods of fixing PHILP [19, 20]. In the present study, we aimed to investigate the effects of contact reduction of inferomedial cortical bone at proximal humerus and placement of calcar screw on the stability of proximal humerus fractures, as well as to address the dynamic and static biomechanics, to provide a theoretical reference for clinical treatment.

## Materials and methods

### Experimental specimens and preparation

**Fig. 1:**
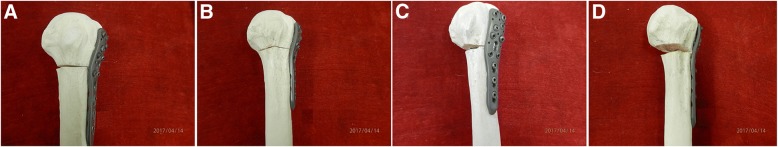
Examples of the Synbone prosthetic humeri as used in each experimental model. The cortex contact + screw fixation group (**a**), the cortex contact group (**b**), the screw fixation group (**c**), and the control group (**d**)

Synbone prosthetic humeri (No. 5010, humeral length 361 ± 4 mm, humeral head diameter 53.0 ± 0.6 mm, SYNBONE-AG, Switzerland) were used in this study. A total of 12 prosthetic humeri were randomized into four groups, each consisting of three prosthetic humeri: the cortex contact + screw fixation group (Fig. 1a), the cortex contact group (Fig. 1b), the screw fixation group (Fig. 1c), and the control group (Fig. 1d). An oscillating saw was used to develop a wedge osteotomy model at the proximal humerus, during which all procedures were conducted manually. At the same time, all the specimens were fixed with the 10-well slotted bone locking plate PHILP (PHILP, Jiangsu Ideal Medical Science & Technology Co., Ltd., China) that was made of titanium, and those in the cortex contact + screw fixation and screw fixation groups were fixed with calcar screws as well and were placed in the position according to the standard rotation and fixation for the surgery (Fig. 1).

All specimens were consistent in height, structure, load, and fixation method and underwent the same mechanical test method, to ensure experimental accuracy. Both ends of the Synbone prosthetic humerus were embedded with denture base resin (Shanghai Medical Instrument Co., Ltd., China) and fixed, and only the bone plate fixation area at the proximal end was exposed. Then, the fixtures at both ends were fixed in parallel to the Zwick/Roell dynamic mechanics tester (Amsler HFD 5100B, Germany). Via the center on the osteotomy section, the relative displacement was measured using a high-precision digital grating displacement sensor (Shanghai University of Science and Technology Instrument Factory, China), with an accuracy of 5 um. After each specimen was carefully mounted, cyclic compression was applied with a loading of 500 N (10 H_Z_) in the axial direction to test the dynamic fatigue properties. The finite fatigue life of each group was measured [21].

For quasi-static mechanical testing, each specimen underwent axial compression testing, three-point bending testing, and torsion testing sequentially. The load and loading rate in the axial compression test were respectively 500 N and 1.50 mm/min, and the load and the maximum bending moment in the three-point bending test were respectively 250 N/min and 7.5 N m; in the torsion test, the load was gradually increased stepwise by 0.6 N m/min to a maximum load of 3 N m. During the loading process, the stress and displacement of the humerus were measured and automatically recorded using the YD-14 dynamic digital resistance strain gauge indicator (Huadong Electronic Instrument, China). In each experiment, the specimen was first pre-loaded with 100 N to eliminate bone relaxation, creep, and other rheological effects. For each loading, the measurement was repeated three times, and each measurement result was used to calculate the mechanical parameters of each group [21].

### Statistical analysis

Statistical analyses were performed using SPSS13.0 software (SPSS, Chicago IL, USA). Data were analyzed using one-way analysis of variance (ANOVA), and intergroup comparisons were conducted using the Student–Newman–Keuls method. A difference with *P* < 0.05 was considered statistically significant.

## Results

### Dynamic mechanical properties of the humerus

As can be seen in Table 1 the cortex contact + screw fixation group showed the longest fatigue life. However, with a fracture face displacement of more than 10 mm, the humeral fracture site successively experienced fracture dislocation and continuous breakage and collapse, which was considered destruction. Meanwhile, refracture of the fracture site, bone piece detachment, slight bending of the calcar screw, askew of the support surface, and breakage due to severe rupture were all found in each of the remaining groups, which resulted in a continuous decline in fatigue life. The finite fatigue life N showed statistically significant differences (*P* < 0.01) of 35%, 43%, and 63% between cortex contact + screw fixation and cortex contact groups, cortex contact + screw fixation and screw fixation groups, as well as cortex contact + screw fixation and control groups, respectively.

**Table 1:** Fatigue mechanics test results of the four groups (mean ± SD)

Type	Cortex contact + screw fixation	Cortex contact	Screw fixation	Control	*P*
Humerus height, *H* (mm)	285 ± 3	282 ± 4	286 ± 4	284 ± 2	1
Head and neck, *φ* (mm)	53.0 ± 0.2	53.0 ± 0.3	53.0 ± 0.4	53.0 ± 0.2	1
Load, *P* (N)	500	500	500	500	1
Frequency (Hz)	10	10	10	10	1
Cycles (N)	6682 ± 401	4338 ± 263	3801 ± 230	2402 ± 172	< 0.01
Destruction morphology	Fracture line rupture	Re-fracture	Slight bending of calcar screw	Severe rupture and break	N/A

The difference in fatigue life between the cortex contact group and the control group was 43–45% (*P* < 0.01). In addition, the difference in fatigue life between the calcar screw fixation group and the control group was 35–37% (*P* < 0.01). These results showed that inferomedial cortical bone contact and fixation with two calcar screws for the treatment of humerus fracture had a very positive effect on the fatigue life, and the fracture instability gradually declined in the remaining three groups: cortex contact + screw fixation group > cortex contact group > screw fixation group > control group.

From the dynamic fatigue mechanics test results, the four groups showed similar-shaped fatigue life curves, which experienced a pattern of deadlock, flattening, decline, increased displacement of the humeral head, continuous deformation and dislocation of the head, and collapse until destruction. When the displacement of the refracture was greater than 10 mm, the PHILP plate loosened, and the calcar screws displayed continuous bending micro-phenomenon until the fracture fixation was breached and detached, so that the whole part collapsed and broke.

### Axial compressive strength and stiffness

**Fig. 2:**
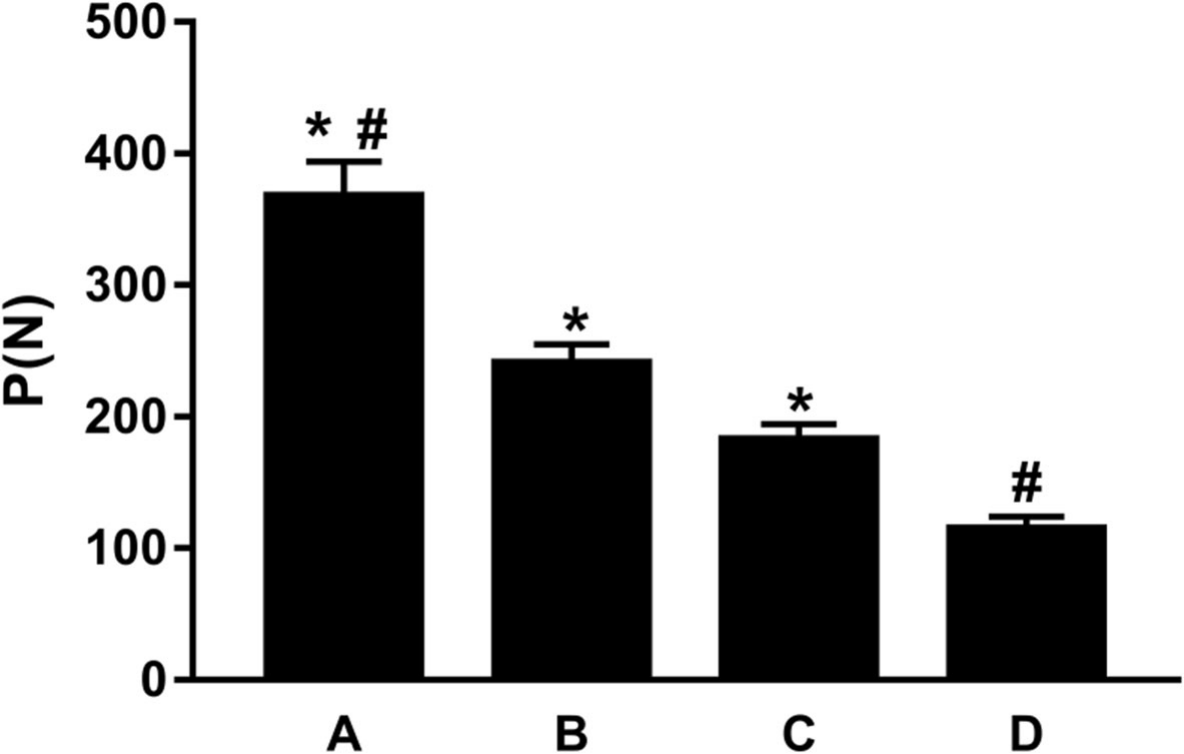
Comparison of the maximum axial compression load among the four groups: the cortex contact + screw fixation group (**a**), the cortex contact group (**b**), the screw fixation group (**c**), and the control group (**d**). Asterisk represents a significant difference compared to; number sign represents a significant difference compared to

With the axial compressive loading, the maximum axial compressive loads of the humerus specimens of the cortex contact + screw fixation, cortex contact, screw fixation, and control groups were respectively (367.41 ± 21.66) N, (240.22 ± 14.46) N, (180.18 ± 12.26) N, and (114.35 ± 9.78) N, where the differences among the four groups were statistically significant, of which the cortex contact + screw fixation group showed the highest load (Fig. 2).

**Fig. 3:**
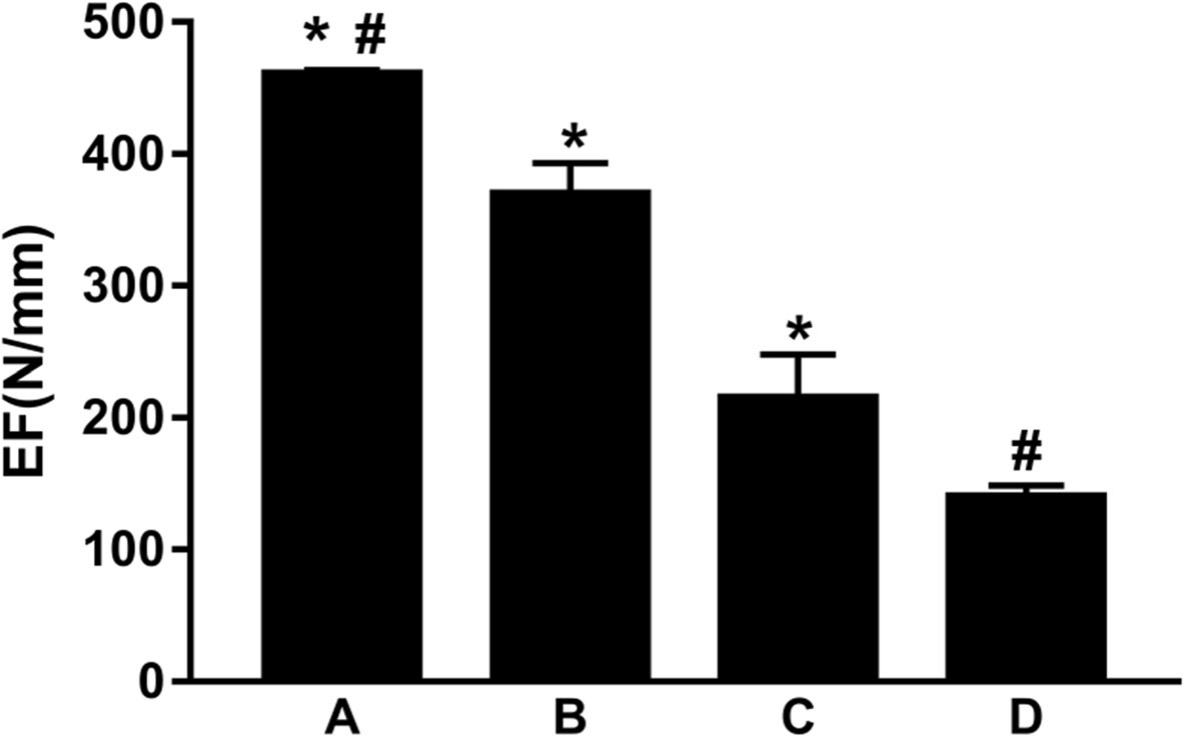
Comparison of the axial compressive stiffness among the four groups: the cortex contact + screw fixation group (**a**), the cortex contact group (**b**), the screw fixation group (**c**), and the control group (**d**). Asterisk represents a significant difference compared to; number sign represents a significant difference compared to

The axial compression stiffness of the cortex contact + screw fixation, cortex contact, screw fixation, and control groups were respectively (460.21 ± 3.22) N/mm, (369.17 ± 23.97) N/mm, (214.73 ± 32.88) N/mm, and (139.98 ± 8.40) N/mm, where the differences among the four groups were statistically significant, of which the cortex contact + screw fixation group had the greatest axial compression stiffness. These results indicate that humeral inferomedial cortical bone contact and fixation with two calcar screws provided the best compressive strength and stiffness and presented the optimal curative effect (Fig. 3).

### Three-point bending moment and bending stiffness

**Fig. 4:**
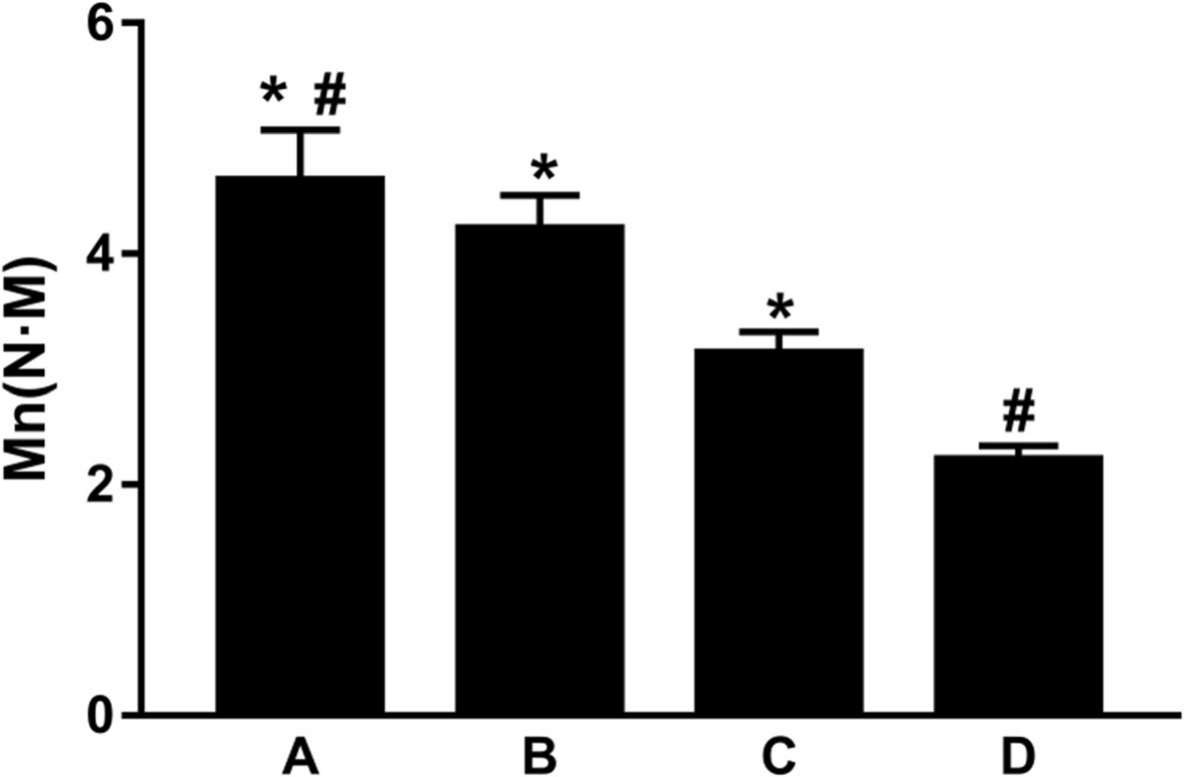
Comparison of the three-point bending moment among the four groups: the cortex contact + screw fixation group (**a**), the cortex contact group (**b**), the screw fixation group (**c**), and the control group (**d**). Asterisk represents a significant difference compared to; number sign represents a significant difference compared to

The bending moments of the cortex contact + screw fixation, cortex contact, screw fixation, and control groups were respectively (4.63 ± 0.44) N m, (4.21 ± 0.44) N m, (3.13 ± 0.19) N m, and (2.21 ± 0.13) N m. Further intergroup comparisons returned statistically significant differences (*P* < 0.01). Meanwhile, the bending stiffness showed similar regularity with the bending moment, and the differences among the groups were statistically significant (*P* < 0.01), of which the cortex contact + screw fixation group showed greatest bending stiffness (Fig. 4).

### Torsion mechanical characteristics

**Fig. 5:**
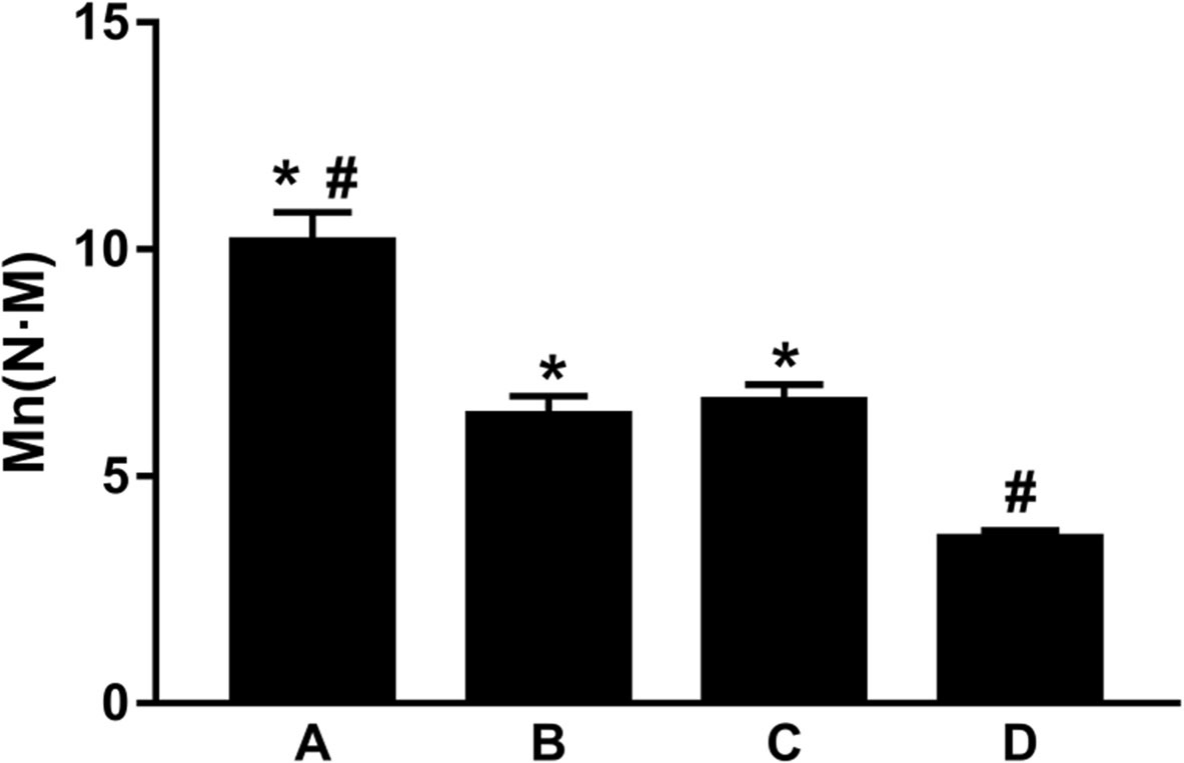
Comparison of the torsion moment among the four groups: the cortex contact + screw fixation group (**a**), the cortex contact group (**b**), the screw fixation group (**c**), and the control group (**d**). Asterisk represents a significant difference compared to; number sign represents a significant difference compared to

The torsion moment and torsion angle changed linearly, that is, the torsion moment increased with increased torsion angle. The maximum torsion moments of the cortex contact + screw fixation, cortex contact, screw fixation, and control groups were respectively (10.16 ± 0.66) N m, (6.33 ± 0.44) N m, (6.64 ± 0.38) N m, and (3.62 ± 0.18) N m, where the differences among the four groups were statistically significant. Further comparison between two groups showed that the difference between cortex contact and screw fixation groups was statistically insignificant (*P* > 0.05), while the maximum torsion moment of the cortex contact + screw fixation group showed a significant difference compared with those of the other three groups (*P* < 0.01, Fig. 5).

**Fig. 6:**
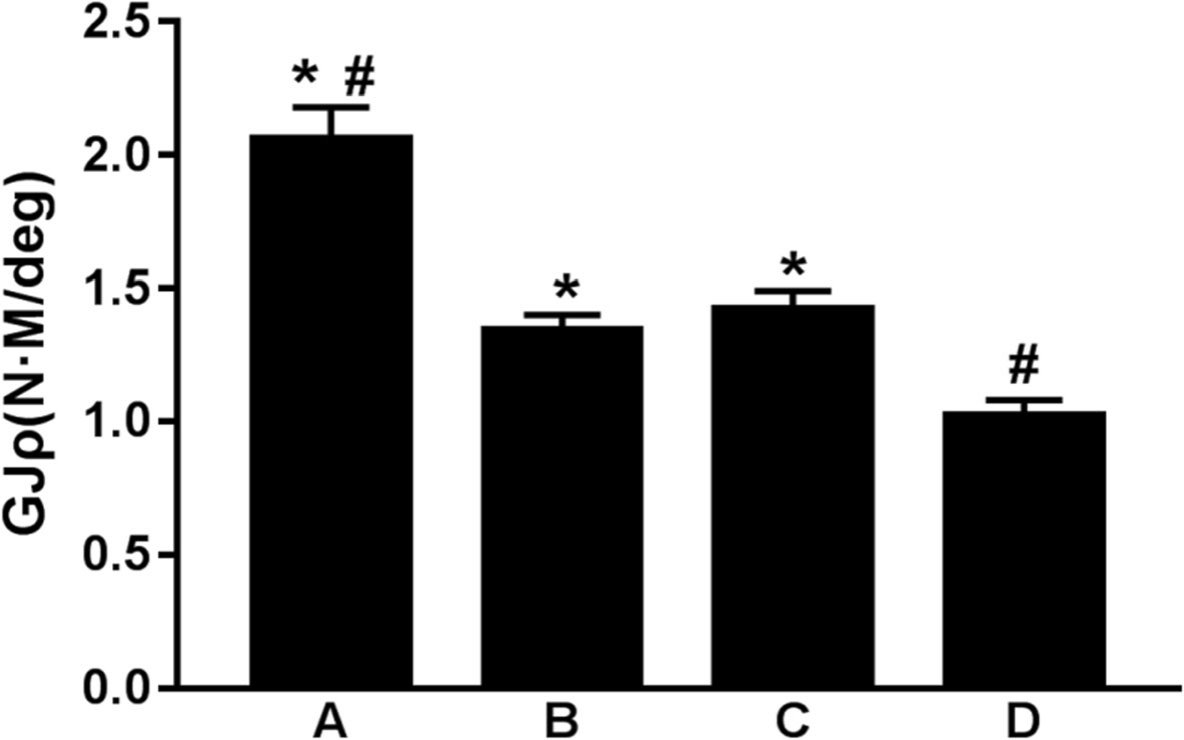
Comparison of the torsion stiffness among the four groups: the cortex contact + screw fixation group (**a**), the cortex contact group (**b**), the screw fixation group (**c**), and the control group (**d**). Asterisk represents a significant difference compared to; number sign represents a significant difference compared to

The torsion stiffness of the cortex contact + screw fixation, cortex contact, screw fixation, and control groups were respectively (2.06 ± 0.12) N m/deg., (1.34 ± 0.06) N m/deg., (1.42 ± 0.07) N m/deg. and (1.02 ± 0.05) N m/deg., where the differences among the four groups were statistically significant (*P* < 0.01). Further comparison between two groups revealed that the difference between cortex contact group and screw fixation group was not statistically significant (*P* > 0.05), but the maximum torsion stiffness of the cortex contact + screw fixation group showed significant differences compared with those of the other three groups (*P* < 0.01, Fig. 6).

## Discussion

The aim of this study was to explore the use of PHILP for the treatment of proximal humerus fractures, in particular, to examine the effect of retaining inferomedial cortical bone contact and fixation with calcar screws on the dynamic and static mechanical stability of the fractures. The results showed that stability, axial compressive load and axial compressive stiffness, bending moment and bending stiffness, and torsion moment and torsion stiffness were all higher in the group that had both cortex contact and calcar screw fixation.

The dynamic fatigue mechanical characteristics after fixation with the locking plate showed that the cortex contact + screw fixation group had the longest fatigue life and the best stability, while the fatigue life was decreased in the other three groups. Further comparison between two groups showed cortex contact + screw fixation group> cortex contact group> screw fixation group> control group. This result is supported by a previous study that also found that in humeri with normal alignment, calcar screws can provide additional stability; however, they also showed that this benefit is lost with varus deformity, but not when a medial deficiency exists [22].

The quasi-static mechanics test revealed that the axial compressive load and axial compressive stiffness of the cortex contact + screw fixation group were 35%, 50%, and 70% higher than the other three groups. Meanwhile, the bending moment and bending stiffness of the cortex contact + screw fixation group were 37%, 53%, and 66% higher than those of the other three groups. The torsion moment and torsion stiffness of the cortex contact + screw fixation group were 37, 35%, and 64% higher than those of the other three groups. Comparison between two groups revealed cortex contact + screw fixation group > cortex contact group > screw fixation group > control group. These results compare well to the results of Zhang et al. [23] who showed that there were similar biomechanical advantages when using medial support screws.

Restoring the inferomedial cortical bone support showed even more advantages in dynamic and static biomechanics compared with placing calcar screws alone for the treatment of humerus fractures. The presence or absence of inferomedial cortical bone contact and the static mechanical indicators accounted for respectively 44% and 40–50% in effect on stability. Meanwhile, placement of calcar screws accounted for 36% and 30% in effects on dynamic and static stabilities, respectively, which indicated that it was more important to restore the inferomedial cortical bone contact, similar to a previous study [23].

In case of comminuted inferomedial cortex and poor reduction, especially in case of the absence of inferomedial cortex support, the screws tend to shift, and even pierce the humeral head, which decreases the stability of the implants. In case of absence of inferomedial cortex support, the incidence of varus deformity cannot be prevented even with increased cement. Restoration of inferomedial cortex support could augment stability and strength of the locking plate and would be expected to reduce the incidence of complications [23]. The presence or absence of inferomedial cortex support is an important indication of whether the reduced fracture will reposition or not. Inserting a locking screw from the lateral side to the middle humeral head cannot play a separate role in maintaining the stability of medial column fractures. Meanwhile, sufficient stability can be achieved by contact of the inferomedial cortex or by the locking plate placed in a particular direction near the inferomedial cortex when the inferomedial cortex is comminuted. To avoid damage to the axillary nerve, some physicians may not insert the calcar screws, especially when using percutaneous fixation techniques because they are concerned this may increase the risk of damage to the axillary nerve. Placement of calcar screws did not increase the risk of damaging the axillary nerve and did not increase the risk of screw cutting and delayed union of the fracture [24]. So far, the evidence suggests that using calcar screws and inferomedial cortex support results in significantly lower complications, but further studies are needed [25].^.^

This study has some limitations; as a synthetic model, the effect of surrounding tissues on these results could not be studied and we could not model the many different complex fractures that are seen clinically. The treatment of comminuted fractures needs further study.

## Conclusions

The results of this study show that proximal humerus fracture fixation retaining the inferomedial cortical bone contact with calcar screw fixation provided better biomechanical characteristics than retaining the inferomedial cortical bone contact or calcar screw fixation alone. Of the two methods, the most important from a biomechanical aspect is retaining inferomedial cortical bone contact.

## Abbreviation

PHILP: Proximal humeral internal locking plate
